# Stress echocardiography in heart failure

**DOI:** 10.1186/1476-7120-2-11

**Published:** 2004-07-30

**Authors:** Eustachio Agricola, Michele Oppizzi, Matteo Pisani, Alberto Margonato

**Affiliations:** 1 Division of Non-Invasive Cardiology, Cardiothoracic Department, San Raffaele Hospital, IRCCS, Milano, Italy

**Keywords:** stress echocardiography, heart failure, diastolic dysfunction, mitral regurgitation

## Abstract

Echocardiography has the ability to noninvasively explore hemodynamic variables during pharmacologic or exercise stress test in patients with heart failure. In this review, we detail some important potential applications of stress echocardiography in patients with heart failure. In patients with coronary artery disease and chronic LV dysfunction, dobutamine stress echocardiography is able to distinguish between viable and fibrotic tissue to make adequate clinical decisions. Exercise testing, in combination with echocardiographic monitoring, is a method of obtaining accurate information in the assessment of functional capacity and prognosis. Functional mitral regurgitation is a common finding in patients with dilated and ischaemic cardiomyopathy and stress echocardiography in the form of exercise or pharmacologic protocols can be useful to evaluate the behaviour of mitral regurgitation. It is clinical useful to search the presence of contractile reserve in non ischemic dilated cardiomyopathy such as to screen or monitor the presence of latent myocardial dysfunction in patients who had exposure to cardiotoxic agents. Moreover, in patients with suspected diastolic heart failure and normal systolic function, exercise echocardiography could be able to demonstrate the existence of such dysfunction and determine that it is sufficient to limit exercise tolerance. Finally, in the aortic stenosis dobutamine echocardiography can distinguish severe from non-severe stenosis in patients with low transvalvular gradients and depressed left ventricular function.

## Background

**Figure 1:**
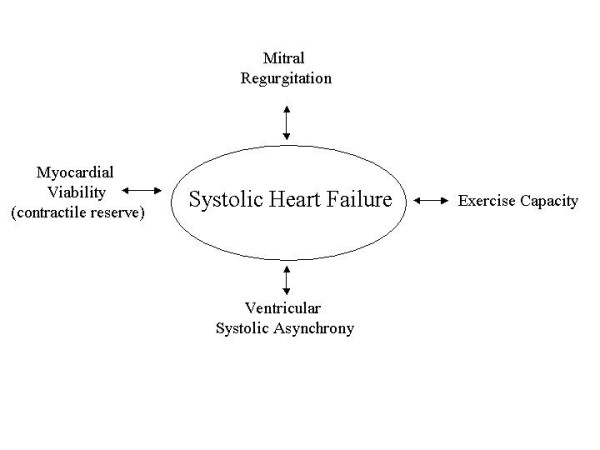
Physiopathologic components of systolic heart failure that can be potentially explored with stress echocardiography.

**Figure 2:**
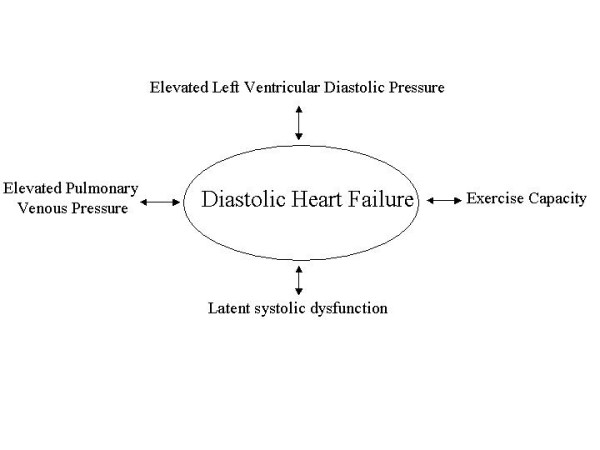
Physiopathologic components of diastolic heart failure assessable with stress echo.

The identification of viable hibernating myocardium in patients with coronary artery disease and chronic left ventricular (LV) dysfunction is, up to today, the most common use of stress echocardiography in patients with heart failure. However, to search viable myocardium or the presence of contractile reserve is only one of plugs of the physiopathologic puzzle in a failing heart (Figure 1 and 2). If we consider the ability of echocardiography to provide valuable haemodynamic information accurately and non-invasively, it is ideally suited for application during stress testing to objectively assess other physiopathologic components of heart failure. These include the study of exercise physiology, the presence and the behaviour of concomitant mitral regurgitation (MR), the prediction of response to resynchronization therapy etc.

Therefore, the present review will detail some important potential applications of stress echocardiography in patients with heart failure in the evaluation of the different clinical and physiopathologic aspects of heart failure syndrome.

### Systolic heart failure

#### Searching the myocardial viability

The most common cause of heart failure in the Western world is coronary artery disease, accounting for up to 60% of cases [1]. In patients with coronary artery disease and chronic LV dysfunction, it is crucial to distinguish between viable and fibrotic tissue to make adequate clinical decisions. Noncontractile but viable myocardium may correspond to different states that are important but difficult to distinguish, i.e., ischemia, stunning, nontransmural infarction, or hibernation and in individual patients these pictures may coexist [2].

After brief episodes of coronary occlusion and reflow a reversible global LV dysfunction can occur. This phenomenon was called myocardial stunning [3]. It is characterized as prolonged mechanical dysfunction after coronary reflow despite resumption of normal perfusion and lack of permanent tissue damage. Stunning seems to result from alterations in contractile proteins in response to sublethal ischemic insults. This phenomenon can occur in several settings, including after acute reperfused myocardial infarction and after CABG. In humans, the return of functional recovery may require days to weeks [4]. Hence, diagnostic methods to distinguish stunning from necrosis are particularly relevant for clinical investigation and management in patients with acute, severe LV dysfunction or cardiogenic shock after revascularization. Persistent wall motion abnormalities can be observed by echocardiography at a time when chest pain, ST segment deviation, and regional perfusion had recovered. The presence of contractile reserve during dobutamine infusion identifies the stunning but viable myocardium from myocardial necrosis.

The term "*Hibernating myocardium*" was first termed by Rahimtoola to indicate the state of reversible dysfunctional myocardium, which was considered to be the result of a state of persistently impaired myocardial function at rest, caused by reduced coronary blood flow, and which could be partially or completely restored to normal either by improving blood flow or reducing oxygen demand [5].

Echocardiography can detect viable myocardium during infusion of drugs which have ability to elicit an enhanced contractile response by recruiting contractile proteins. At least two drugs have these proprieties: the dobutamine, a synthetic β1 agonist with additional α1- and β2-stimulating properties and the enoximone that inhibits cyclic adenosine monophosphate-specific phosphosdiesterase [6,7]. Routinely, the dobutamine is the most common stressor used, whereas the enoximone is particularly useful in patients on beta-blocker therapy [7,8]. The mechanism by which dobutamine stimulation elicits a contractile response in hypoperfused dysfunctional segments without precipitating ischemia has been demonstrated by Sun et al. [6]. By using positron emission tomography and echocardiography, they demonstrated that the improvement in contractile function during dobutamine infusion was associated with a concomitant increase in myocardial blood flow. The increase in myocardial blood flow occurs because there is persistent, albeit reduced, coronary flow reserve distal to a stenosis which dobutamine may exploit. Another mechanism whereby contractile response may be elicited during dobutamine infusion is through its peripheral vasodilator effect, which causes reduction in LV end-systolic wall stress by reducing afterload [6]. Moreover, dipyridamole echocardiography (up to 0.84 mg/kg over 10 minutes) can identify regions with myocardial viability [9]. Dipyridamole leads to transiently increased coronary flow, which leads to improved contractility in viable myocardium [9]. A small study comparing dipyridamole with dobutamine revealed 93% concordance [10]. Combined dipyridamole-dobutamine (low-dose dipyridamole followed by low-dose dobutamine) has also been proposed and found to recruit a contractile reserve in some asynergic segments that were nonresponders after dobutamine or dipyridamole alone [11].

An initial evaluation of end diastolic wall thickness of akinetic segments with resting echocardiography can be used as an initial screening technique for assessment of viability. Indeed, akinetic regions with an end diastolic wall thickness <6 mm do not contain viable myocardium and do not improve in function after revascularization [12]. However, in segments with a thickness ≥ 6 mm, additional testing is needed because approximately 40% of these regions do not contain viable myocardium and will not improve after revascularization [12]. Therefore, myocardial thinning should not be equated with the lack of myocardial viability, and in some patients, these regions can improve in contractile function after revascularization [13]. The detection of subendocardial infarcts became clinically relevant because the quantification of non-viable myocardium in addition to viable myocardium in that region of LV is important in predicting contractile improvement following revascularization. Thus, the ratio of viable to total myocardium (viable plus non-viable) in the dysfunctional region was more accurate that absolute amount of viable myocardium alone in predicting functional improvement [13]. Unfortunately, currently available techniques, such as single photon emission computed tomography, dobutamine stress echocardiography and positron emission tomography are still insufficient to provide a comprehensive assessment including the evaluation of subendocardial infraction with respect to magnetic resonance imaging [14].

During stress echocardiography is possible to observe four response patterns based on regional wall function: normal, ischemic, viable and necrotic. In the normal response, a segment is normokinetic at rest and normal or hyperkinetic during stress. In the ischemic response, a segment worsens its function during stress from normokinesis to dyssynergy. In the necrotic response, a segment akinesia remains akinetic during stress. In the viability response, a segment with resting dysfunction improves during stress. During pharmacologic stress, a viable response at low dose can be followed by ischemic response at high dose (biphasic response). This biphasic response is suggestive of viability and ischemia, with jeopardized myocardium fed by a critically stenosed coronary artery [15]. A resting akinesia which becomes dyskinesia during stress reflects a purely passive mechanical phenomenon and should not be considered a true active ischemia.

The overall sensitivity and specificity of dobutamine echocardiography for predicting recovery of *regional function *after revascularization was 84% and 81% respectively [16]. In a study by Afridi et al., the best predictive value for recovery of function after revascularization was most often noted in patients demonstrating an ischemic response during low and high doses of dobutamine infusion [17]. On the other hand, sustained improvement of regional function during dobutamine infusion was a poor marker of recovery function.

Sensitivity of dobutamine echocardiography may be affected by several factors such as the severe reduction of myocardial blood flow that can preclude the contractile response, the premature interruption of dobutamine infusion, resting tachycardia that may renders the myocardium ischemic and dobutamine can only augment ischemia [16]. On the contrary, the specificity may be affected by the tethering effect, the injured subendocardial portion of myocardium that does not respond to dobutamine when the infarction is confined to subendocardium, and also specificity may be reduced in myocardial regions that do not develop an ischemic response [16].

**Figure 3:**
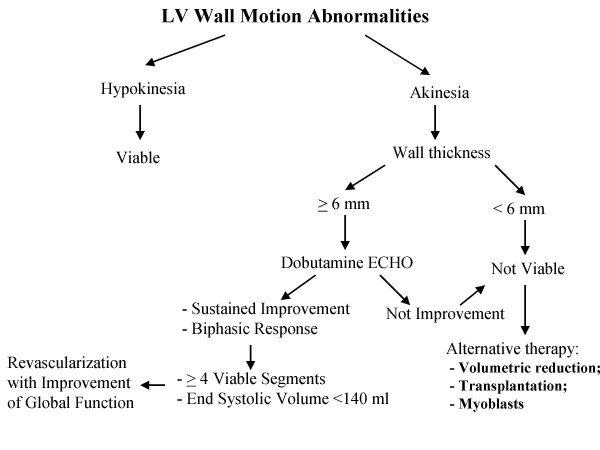
A schematic flow chart for searching segmental and global systolic function in chronic ischemic LV dysfunction.

The main clinical issue to search the myocardial viability is that patients with evidence of hibernating myocardium who do not undergo revascularization have poor prognosis with high incidence of cardiac events [18]. In contrast, evidence of viable myocardium in patients undergoing successful revascularization is associated with longer survival and improvement of both symptoms and LV function [19]. However, the presence of myocardial viability is only relevant in patients with severely depressed LV function and has a prognostic impact only when a significant amount of viable myocardium is present. Therefore, the final end point of searching the myocardial viability is to predict the recovery of *global myocardial function *after revascularization. At this purpose there is a relation between improvement in left ventricular ejection fraction (LVEF) and the number of segments with contractile reserve, indicating that extent of jeopardized but viable myocardium determine the magnitude of improvement of LV function after revascularization. Usually a level of ≥ 4 viable segments, which corresponds an improvement in wall motion score index >0.25 (about 20% of left ventricle), is advised as a cutoff value to predict improvement of LVEF [20]. However, despite the presence of substantial viability, in some patients LVEF does not improve after revascularization because not only the amount of dysfunctional but viable tissue but also LV remodelling and enlargement determines the improvement in function following revascularization [21]. Thus, patients with a high end systolic volume (≥ 140 ml) due to LV remodelling have a low likelihood of improvement of global function [21] (Figure 3).

#### Assessing the functional capacity

In most patients with chronic heart failure, symptoms are not present at rest but become limiting with exercise. Despite this, the major measures used to characterise the symptoms, the severity, the mechanisms and the prognosis of heart failure are obtained at rest. Exercise testing, in combination with echocardiographic monitoring, may be an attractive and practical method of obtaining accurate information which can aid in the diagnosis of heart failure as well as the assessment of functional limitation and prognosis. Exercise rather than dobutamine is the stressor of choice to evaluate functional capacity due to the possibility to combine echocardiographic variables with common parameters available during physiologic exercise.

Symptom limited exercise testing can be undertaken using either treadmill or bicycle exercise protocols. Available data about the safety of exercise testing in patients with significant heart failure suggest a very low incidence of serious adverse events such as arrythmias or hypothension.

**Figure 4:**
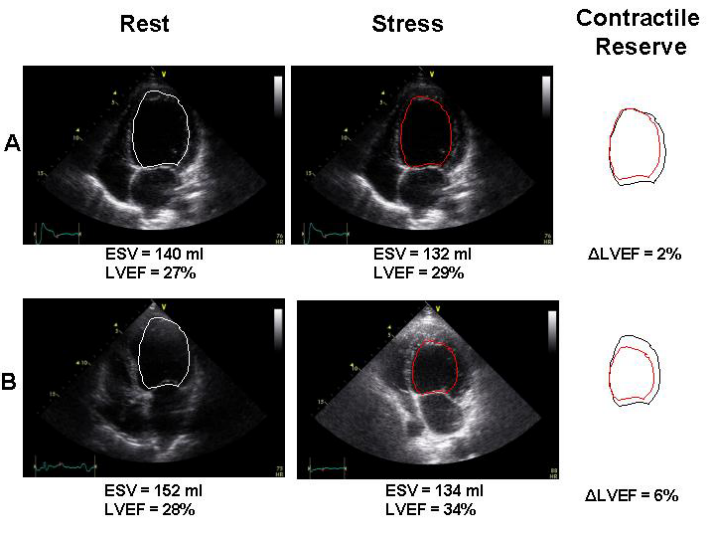
Echocardiographic apical four-chamber images (end-systolic frames) from two patients with and without contractile reserve.

The echocardiographic monitoring during exercise testing may have an additional value overt the conventional parameters assessed during exercise testing such as functional capacity, symptoms and peak oxygen uptake that become part of the final interpretation. Indeed, several haemodynamic parameters can be noninvasively obtained with echocardiography such as LVEF at rest and during stress deriving the contractile reserve, the behaviour of mitral valve function, the pulmonary artery pressure, the right ventricular function, the diastolic function (Table 1). In this way, it is possible to observe the variation of the monitored variables and to correlate these with the appearance of symptoms, i.e. impairment of global contractile function followed by increase in pulmonary pressure with dyspnoea. The critical level to define the presence of contractile reserve is generally defined as an increase of at least 5% (in absolute terms) in the global LVEF [22] (Figure 4). The change in the systolic pulmonary artery pressure (sPAP) at rest and during exercise is among others, the most frequently utilized echocardiographic variable. It can reliably be estimated by adding the right atrial pressure derived from the tricuspid reguritation jet velocity [23]. The right atrial pressure can be estimated at rest by the response of inferior vena cava to deep inspiration and assumed to be constant throughout exercise. Sometimes the use of echocardiographic contrast agents such as agitated saline solution may help to enhance Doppler signals. Pulmonary hypertension determined by echocardiography has been defined as a peak of sPAP >30 mmHg at rest and >45 mmHg during exercise [24]. Right ventricular dysfunction predicts impaired exercise capacity and decreased survival in patients with both moderate and advanced heart failure [25]. There are several clinically validated methods to detect right ventricular dysfunction. Tricuspid annular plane systolic excursion (TAPSE) visualized from the apical four-chamber view is an easy measure and can be used a surrogate of right ventricular function. A TAPSE value of 14 mm or less means the presence of right ventricular dysfunction and is a significant adverse prognostic indicator [26]. More recently, the evalution of tricuspid systolic annular tissue Doppler velocity has been introduced as index of right ventricular function and a value less than 10.8 cm/s indicates patients with abnormal right ventricular function [27].

**Table 1:** Potential parameters obtainable during exercise echocardiography.

**Common variables during exercise test**	**Additional echocardiographic variables during exercise test**
Duration of exercise	Contractile reserve
Peak VO2	Mitral valve function
Anaerobic threshold	Pulmonary systolic pressure
Oxygen pulse	Right ventricular function
VO2 workload ratio	Diastolic function
O2 saturation	

Moreover, these evaluations are useful not only for the diagnosis but also for predicting the outcome of patients overt the symptoms of heart failure. Indeed, some patients with marked reduction in myocardial contractility at rest, but with good residual contractile reserve, have a favourable exercise capacity and prognosis, whereas patients with mild symptoms and similar degree of abnormal myocardial contractility at rest, but without contractile reserve, have poor outcome [28]. Although a VO2max <14 ml/kg/min is well known as a measure for deciding on eligibility for cardiac transplantation, it has been clearly shown that there is no absolute threshold for adverse prognosis and that VO2max uptake should be considered as a continuous variable. In term of discriminating survivors from non survivors, it appears that VO2max <10 ml/kg/min definitively defines high risk, while a value >18 ml/kg/min defines low risk; those values in between may represent a grey zone. Thus stress echocardiography yields the greatest incremental prognostic value in patients with intermediate values of VO2max (10–14 ml/Kg/min) and helps to further stratify the risk of patients with intermediate (Table 2) [29].

**Table 2:** Additive prognostic value of stress echo in patients with intermediate values of VO2max (10–14 ml/Kg/min).

**Risk**	**Low (5–10% year)**	**High (≥ 25–30% year)**
Exercise capacity	≥ 8–10 min	<8 min
Contractile reserve	yes	no
Pulmonary hypertension	<45 mmHg	>45 mmHg
Right ventricular dysfunction	no	yes
Mitral regurgitation	↓ or =	↑↑

#### Looking at the behaviour of mitral valve

MR is a common finding in heart failure patients. In patients with dilated and ischaemic cardiomyopathy, the MR is typically functional and reflects geometric distortions of LV chamber, which displaces the normal valve and subvalvar closing mechanisms. This functional MR is a consequence of adverse LV remodelling and increased sphericity of the chamber. It is typically dynamic and a marker of adverse prognosis. The 5-year survival of heart failure patients with functional MR ranges from 39.9% to 48.7% depending on the degree of MR [30].

Stress echocardiography in the form of exercise or pharmacologic protocols can be useful in the assessment of MR. Exercise echocardiography is usually preferred due to the possibility to reproduce physiological setting. Even though supine bike protocol allows to obtain good image acquisition, upright bicycle or treadmill protocols are more frequently utilized in the practical setting. Treadmill exercises can be performed using the standard protocols such as Bruce or modified Bruce, while gradual increase in the bike workload of 20–25 W every 2–3 minutes is often applied until the patients achieves either the target heart rate or develops symptoms of fatigue or shortness of breath. Sometimes, pharmacologic stress is used with dobutamine protocol at low or intermediate doses infusion. In the collection of echocardiographic data should be included: the MR jet to evaluate the MR jet area and the vena contracta width, the velocity time integrals (mitral and aortic) to calculate the regugitant volume and the effective regurgitant orifice area (EROA), the LV volumes to assess the myocardial contractility and the tricuspid regurgitant jet velocity to measure the sPAP that is an useful index of the haemodynamic burden of MR.

**Figure 5:**
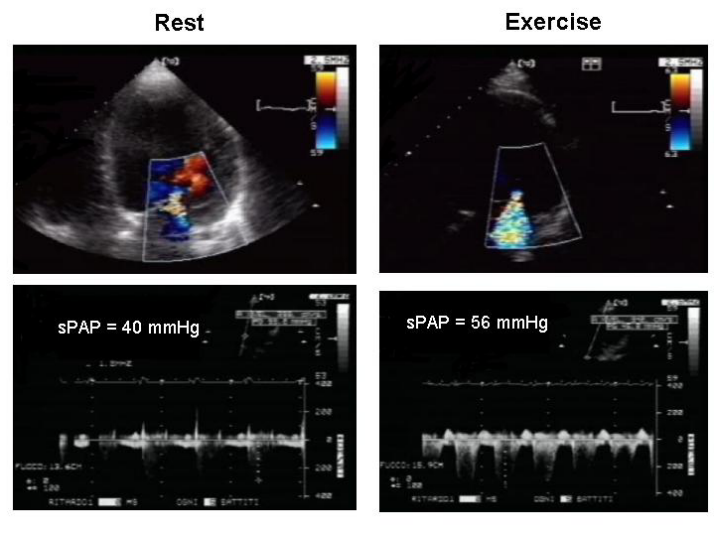
Apical four-chamber view at rest and during exercise in patients with ischemic mitral regurgitation showing a large exercise-induced increase in mitral regurgitation. SPAP = Systolic pulmonary pressure.

**Figure 6:**
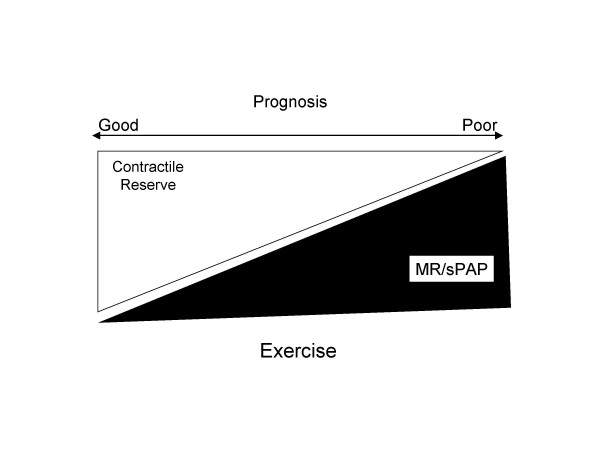
Relationship between contractile reserve, mitral regurgitation and pulmonary pressure and its contribution in defining the prognosis in patients with functional mitral regurgitation. MR = mitral regurgitation; sPAP = systolic pulmonary pressure.

Exercise echocardiography can play several roles in the assessment of the behaviour of mitral valve in heart failure patients. *First*, in symptomatic patients with LV dysfunction and a clinical picture suspicious for new or worsening MR, but not evident at resting echo examination, exercise echocardiography can demonstrate a worsening of MR which helps to correlate this scenario with the patient's symptoms (Figure 5) [31]. *Second*, LV contractility, in presence of MR, can impair or improve during exercise with consequent modification of MR. Patients with presence of contractile reserve show a decrease in MR [32], whereas generally a fall in stroke volume is associated with an increase in mitral regurgitant volume during isometric exercise, which increases systemic resistances and thereby afterload [33]. These observations support the concept of presence of a vicious circle between LV function and behaviour of MR. Therefore, to study these patients with exercise echocardiography may be important for assessing the response of MR to medical therapy and for the following prognostic implications. Indeed, *third*, in patients with ischemic MR and LV dysfunction, quantitative assessment of exercise-induced changes in the degree of MR provides independent prognostic information. Significant exercise-induced increases in MR (increase in ERO ≥ 13 mm^2 ^) unmask patients at high risk of poor outcome. The cardiac mortality rate of medically treated patients with dynamic MR during exercise is 39% at 20 months which represents excess mortality in patients in functional class II or III (Figure 6) [34].

**Figure 7:**
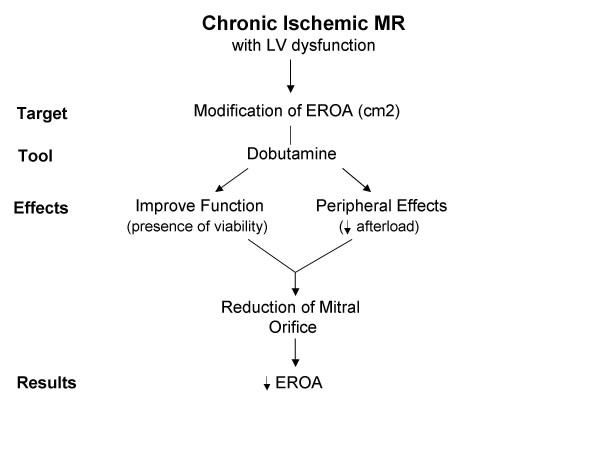
Targets and effects of dobutamine stress echo in patients with mitral regurgitation and chronic ischemic left ventricular dysfunction. EROA = Effective regurgitant orifice area.

Finally, dobutamine protocol has a different role in the contest of ischemic MR. Generally, in this setting it is used to evaluate the behaviour of MR in relation with the presence or absence of myocardial viability (Figure 7). Dobutamine infusion has the ability to decrease MR volume due to a reduction of afterload and mitral orifice size that may occur as a result of the vasodilatory and inotropic effects of dobutamine [35,36]. Therefore, if during dobutamine protocol we find myocardial viability and a concomitant reduction of MR, these results should be interpreted with caution because we cannot assume a direct effect of the presence of myocardial viability on the MR. Thus, the complex interplay between haemodynamic effects of dobutamine, myocardial viability and behaviour of MR has to be taken in mind during clinical management of patient with LV dysfunction and ischemic MR, i.e. revascularization alone versus revascularization plus mitral valve surgery.

#### Evaluating the contractile reserve beyond hibernating myocardium

It is commonly believed that the assessment of contractile reserve is only confined and clinically useful to search the myocardial viability in patients with LV dysfunction and coronary artery disease. Growing published data suggest the utility in searching the presence of contractile reserve in non ischemic dilated cardiomyopathy (DCM). While in the ischemic cardiomyopathy the search of myocardial viability is focused to find the presence of reversible segmental myocardial dysfunction and its possible effect on global systolic LV recovery after revascularization, in DCM the primary end point is to evaluate the presence of residual global contractile reserve. Both dobutamine and exercise testing have been used in the study patients with DCM, but there is a clear predominance for the use of dobutamine test. The doses of dobutamine utilized vary from investigators, but safety in its use in this population has been documented in doses as high as 40 μg/kg per minute. In the interpretation of results both wall motion score index and the LV volume to derive LVEF must be calculated.

LV systolic function at the time of diagnosis has been proposed to be the strongest predictor of survival in DCM, but now the presence of contractile reserve recognised by dobutamine echocardiography seems to be the best marker of good prognosis in patients with severe LV dysfunction at rest [37,38]. Patients with significant improvement in their wall motion score index and LVEF during dobutamine infusion have a better survival rate and increase in the LVEF during follow-up period [37]. The data extracted from dobutamine study can be used as an adjunct or alternative to predict VO2max and exercise capacity of patients with heart failure, especially when the patients fall into the gray zone of VO2max (10–14 ml/kg/min) or when there is limitation to ambulation [29]. Moreover, the response to dobutamine infusion predicts the improvement in LVEF with beta-blocker therapy in patients with advanced heart failure. Patients with contractile reserve experienced a greater improvement in LVEF with beta-blocker by biologically augmenting myocyte a chamber contractility [39]. Whereas, in the absence of contractile reserve (when myocytes have been replaced by fibrosis), ventricular function cannot improve by this biological mechanism because there are not enough contractile units and the sympatholytic effects of beta-blocker prevail [39]. However, the clinical use of dobutamine stress echocardiography in patients with chronic heart failure may be limited by a substantial proportion of patients already receiving beta-blocker therapy at time of evaluation. In these patients enoximone echocardiography might be a valid alternative to low-dose dobutamine for evaluating contractile reserve showing a more potent and a better safety profile than dobutamine [8].

Stress echocardiography may also help in the identification of patients in the initial phase of cardiomyopathy overt normal resting echocardiographic parameters. Both dobutamine and exercise have to be used to screen for the presence of latent myocardial dysfunction in patients who had exposure to cardiotoxic agents [40].

### Diastolic heart failure

The prevalence of diastolic heart failure in the community is now to be at least as high as that reported in previous studies of hospitalised patients; almost half of all patients with heart failure have diastolic heart failure [41]. The term asymptomatic diastolic dysfunction is used to refer to an asymptomatic patient with a normal LVEF and abnormal echo-Doppler pattern of LV filling; this is often seen, for example, in patients with hypertensive heart disease. If such patients exhibit symptoms of effort intolerance and dyspnoea, especially if there are evidence of venous congestion and edema, the term diastolic heart failure can be used [42].

Resting echocardiography is most useful in the assessment of LV size, LVEF and the use of Doppler-derived indices of diastolic function has impact on the identification of diastolic dysfunction. However, to determine whether an abnormality of diastolic function is the cause of the patient's symptoms, we need to demonstrate the existence of such dysfunction and determine that it is sufficient to limit exercise tolerance. Therefore, the stress echocardiography, in particular exercise echocardiography could be useful in dyspnoeic patients with apparently normal LV function to unmask the presence of diastolic dysfunction (signs of elevated LV filling pressure) during exercise as cause of symptoms.

Patients with diastolic heart failure, as well as those with diastolic dysfunction and little or no congestion, exhibit exercise intolerance for several reasons. First, an elevated LV diastolic and pulmonary venous pressure during exercise causes reduction in lung compliance, which increases work of breathing and evokes the symptom of dyspnoea [42]. Second, a substantial number of patients who have LV hypertrophy, high relative wall thickness and small end diastolic volume exhibit a low stroke volume and a depressed cardiac output [43]. These hearts exhibit a limited ability to utilize the Frank-Starling mechanism during exercise. Such limited preload reserve, specially if coupled with the chronotropic incompetence limits the cardiac output during exercise [44]. Third, elevated LV diastolic and pulmonary venous pressures in patients with normal LVEF are directly related to abnormalities in the diastolic proprieties of the ventricle. This is not to say contractile function is entirely normal, but subtle and latent abnormalities of contractile function could be present in many patients, in whom, however, diastolic dysfunction is the dominant feature [42].

**Figure 8:**
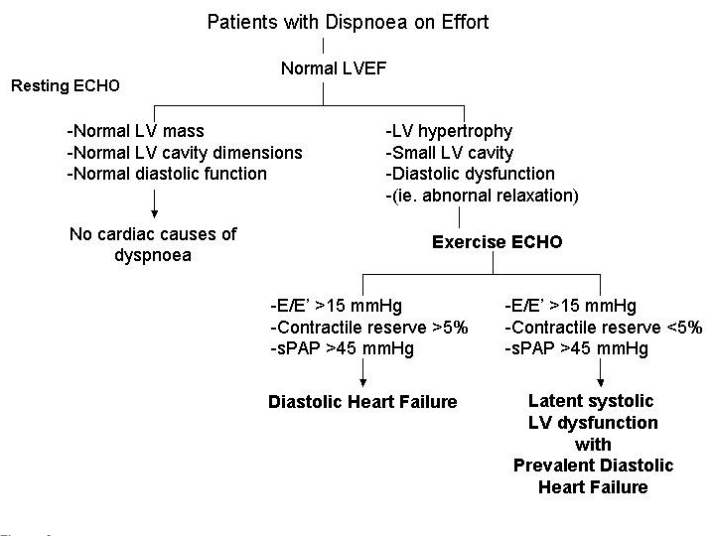
Schematic diagnostic algorithm in patients with suspected diastolic heart failure. LV = Left ventricle; LVEF = Left ventricular ejection fraction.

All these aspects can be assessed during exercise echocardiography (Table 3). In particular the assessment of diastolic function during exercise has been shown to be feasible [45]. Combining transmitral flow velocity with annular velocity obtained at level of the mitral annulus with tissue Doppler (E/E') has been proposed as a tool for assessing LV filling pressures that combines the influence of transmitral driving pressure and myocardial relaxation [46]. Patients with rest E/E' >15 can be classified as having elevated filling pressure. A rest E/E' <8 suggests normal filling pressure and a range of 8 to 15 represents a gray zone. E and E' velocities increased significantly after exercise. In normal subjects because of proportional increases of both velocities, the E/E' ratio do not change significantly with exercise; this observation can be taken as a normal diastolic response during exercise [45]. Indeed, if E/E' ratio increases up to 15 we can suppose a pathological increase of LV filling pressure during exercise. This evaluation must be associated to the assessment of cardiac output and sPAP during exercise with appearance of symptoms. Finally, with the evaluation of systolic LV function during exercise it is possible to discover the portion of patients with concomitant latent myocardial dysfunction but predominant diastolic abnormality (Figure 8).

**Table 3:** Useful echocardiographic parameters to evaluate diastolic function during exercise test in patients with suspected diastolic heart failure.

Transmitral Doppler indices
E/E' ratio
Cardiac output
Preload reserve
Contractile reserve
Pulmonary systolic artery pressure

### Aortic stenosis with left ventricular dysfunction

Stress echocardiography with dobutamine infusion is particularly useful in clinical decision making in patients with aortic stenosis with LV dysfunction and low transvalvular gradients. In this group of patients, an important clinical question rises: is the low gradient a consequence of low cardiac output due to a severe aortic stenosis which has led to LV dysfunction or is the low gradient a consequence of LV dysfunction is unrelated to aortic stenosis and the aortic stenosis is an incidental finding?

It is well known that the transvalvular gradients are flow-depentent parameters so that they are influenced by LV function. The aortic valve area can be accurately determined by Doppler echocardiography with continuity equation and that correlate well with Gorlin formula [47]. However, it has been shown that valve areas calculated by the Gorlin formula is flow-dependent and usually increase with flow, probably due to the flow dependence of the empirical constant C of the Gorlin formula, which represents the ratio of effective to anatomical orifice area. Burwash et al., with dobutamine stress-echocardiography, demonstrate a flow-dependent increase in actual orifice aortic valvular area calculated with continuity equation [48].

**Figure 9:**
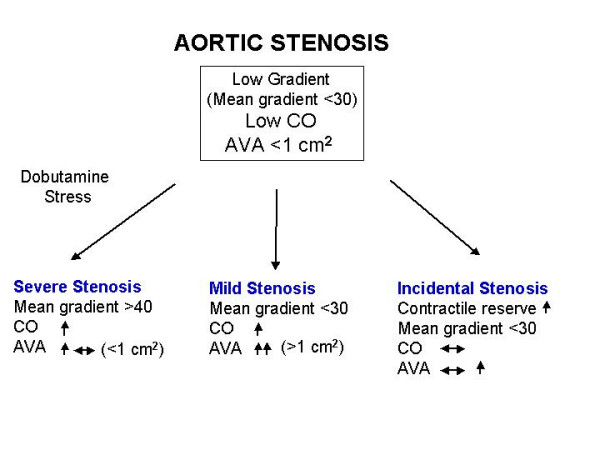
Possible results during dobutamine stress echocardiography in presence of aortic stenosis, low cardiac output and low transvalvular gradients. AVA: Aortic valve area; CO: Cardiac output.

Therefore, the assessment of valve area does not solve the diagnostic dilemma in these patients, because we cannot distinguish between severe fixed from flow-dependent (relative) aortic stenosis. Thus, it is important to perform pharmacological manoeuvres to increase cardiac output so that valve area can be calculated at higher flow rate. Dobutamine stress echocardiography until 20 γ/kg/min with concomitant evaluation of cardiac output, aortic valve area and gradients, is a useful and reliable test to distinguish between severe fixed from relative aortic stenosis in presence of low gradient and LV dysfunction. On the basis on test results, it is possible to distinguish 3 groups of patients [49] (Figure 9): 1. Patients with an improvement of contractile function but no significant increase in valve area and an increase of transvalvular gradients. These patients have severe fixed aortic stenosis and are good candidate for surgery with an acceptable peri-operative surgical risk. 2. Patients with contractile reserve with an increase of aortic valve area without substantial increase in transvalvular gradients. These patients have a non critical aortic stenosis and the LV dysfunction is not related to the aortic stenosis and should be treated conservatively. 3. Finally, patients without contractile reserve and no modification of valve area and transvalvular gradients. These patients represent an ambiguous group, because can represent patients with end-stage severe aortic stenosis with severe LV dysfunction or patients with severe LV dysfunction without contractile reserve and incidental aortic stenosis. However, this group has very poor prognosis.

When interpreting the results of a dobutamine study in these patients to rule out or confirm definitively the presence of a severe fixed aortic stenosis, it is advisable to use an absolute cut-off value of the aortic valve area at peak of dobutamine >1 cm^2 ^rather than an increase of ≥ 0.3 cm^2 ^from baseline alone [49,50].

## Conclusions

Beyond the identification of viable hibernating myocardium, stress echocardiography is particular useful in patients with systolic and diastolic heart failure to assess the different physiopathologic component of heart failure syndrome and can aid to an appropriate clinical decision making.

## List of abbreviations

**LV**: left ventricular

**MR: **mitral regurgitation

**LVEF: **left ventricular ejection fraction

**DCM: **dilated cardiomyopathy

**TAPSE**: Tricuspid annular plane systolic excursion

**sPAP**: Systolic pulmonary artery pressure

## Competing interests

The manuscript is not under consideration elsewhere and the data presented have not been previously published. All authors have read and approved the manuscript. No financial support was received for this study. The content of this manuscript is not associated with any financial interest or other relations that could lead to a conflict of interest.

## Author's contribution

Concerning the authorship, the listed authors have contributed as follows to the manuscript:

EA and MP: 1) conception, design, analysis and interpretation of data, 2) drafting of the manuscript and 3) final approval of the manuscript

MO and AM: 1) critical revision of the manuscript for important intellectual content, and 3) final approval of the manuscript.
